# Machine learning for improved path loss prediction in urban vehicle-to-infrastructure communication systems

**DOI:** 10.3389/frai.2025.1597981

**Published:** 2025-07-11

**Authors:** Mongi Ben Ameur, Jalel Chebil, Mohamed Hadi Habaebi, Jamel Bel Hadj Tahar

**Affiliations:** ^1^ENISO, NOCCS Laboratory, University of Sousse, Sousse, Tunisia; ^2^ISTLS, NOCCS Laboratory, University of Sousse, Sousse, Tunisia; ^3^Department of Electrical and Computer Engineering, International Islamic University, Kuala Lumpur, Malaysia

**Keywords:** path loss modeling, vehicle-to-infrastructure (V2I), path loss prediction, machine learning (ML), XGBoost, multilayer perceptron (MLP), 3GPP model, dual slope model

## Abstract

Path loss prediction is crucial to facilitate reliable vehicle-to-infrastructure (V2I) communications. In this study, machine learning techniques are investigated for path loss modeling using empirical measurements at 5.9 GHz from eight Road Side Unit (RSU) sites. The performance of Extreme Gradient Boosting (XGBoost) and Multilayer Perceptron (MLP) models is contrasted with traditional empirical models such as the Dual Slope and 3rd Generation Partnership Project (3GPP) models in three varied urban environments: open, suburban, and densely urbanized cities. The findings indicate that machine learning models, in particular XGBoost, consistently outperform traditional models with the lowest Root Mean Square Error (RMSE) in complicated urban environments. For additional robustness in prediction, we propose an innovative environmental classification system based on building density, street geometry, and transmitter position. Feature importance examination reveals that distance, environmental class, and transmitter height are the most significant factors affecting path loss prediction accuracy. These observations aid the development of adaptive V2I communication systems and provide valuable guidelines for enhancing reliability in diverse urban environments.

## 1 Introduction

V2I communication is a critical aspect of Intelligent Transportation Systems (ITS). It enables vehicles to communicate with roadside equipment in real-time to make traffic safer, more efficient, and more effectively managed. One of the major issues with deploying trustworthy V2I systems is estimating path loss reliably. Path loss is the extent to which signal intensity weakens as it propagates through urban spaces. Obstacles such as buildings, terrain irregularities, and dynamic objects (e.g., traffic and weather) cause non-linear interference patterns, whose effect on signal reliability is significant. Traditional path loss models, such as the Dual Slope and 3GPP models (Kenney, 2011), are commonly empirical or deterministic in nature. While they work effectively in controlled settings, such models fail to capture the complexity of urban areas, resulting in predictions that can deteriorate V2I communication performance.

Recent developments in machine learning (ML) offer a hopeful alternative. With big data and better learning methods, ML models can learn subtle relationships between environmental factors and signal loss that go beyond the predictive capability of traditional models. XGBoost is a gradient-boosting tool that is good at managing complex data and can work with many variables. It has shown great accuracy in predicting numbers. Likewise, MLP neural networks can learn complicated patterns well, which makes them suitable for predicting V2I path loss. Nevertheless, the performance of such models is highly dependent on effective data preprocessing in the form of outlier elimination, feature scaling, and dataset representativeness to ensure generalizability across various urban settings. This paper contrasts the performance of XGBoost and MLP with Dual Slope and 3GPP benchmarks for urban V2I path loss prediction. With a higher solution set of real-world measurements, i.e., Received Signal Strength Indicator (RSSI), transmission power, and transceivers’ distances, we show that machine-learning models generalize far better than traditional methods. Our findings point out that XGBoost can decrease prediction errors by as much as 38%, which makes V2I link budgeting and network planning more precise. Further, we emphasize preprocessing techniques, considering that better data quality means higher model reliability. These findings have deep implications for ITS adoption in smart cities. Proper path loss prediction is crucial for optimal placement of infrastructure, ensuring reliable vehicle-to-infrastructure communication, and enhancing the performance of autonomous vehicle technologies. This work helps create smart transportation networks by using machine-learning insights in V2I system design. These networks can adapt and respond to the challenges of moving around in today’s cities.

## 2 Previous studies

This section presents existing research on path loss prediction methods, including traditional models, artificial intelligence-based approaches, and deep learning techniques.

### 2.1 Traditional path loss prediction

Traditional path loss prediction models can be broadly divided into two classes: deterministic and statistical. Deterministic models, such as ray tracing, rely on physical propagation equations to make very accurate predictions. They require, however, extensive environmental data and significant computational resources, and they are difficult to scale (Hoomod et al., 2018; Akpaida et al., 2018).

Statistical models, including the COST 231-Hata and ECC-33 models, predict path loss trends from empirical data. While being of useful real-world relevance, they generally lack site-specific accuracy (Tarhuni and Ouni, 2022; Zhang et al., 2023). As established by Rappaport (2002) in his foundational work on wireless communications, these statistical models provide a practical compromise between accuracy and implementation complexity for large-scale deployment scenarios.

For 5.9 GHz city-wide V2I communications of critical significance to intelligent transportation systems (Boban et al., 2011), path loss models must be capable of addressing significant challenges presented by dynamic obstacles (e.g., cars, buildings, and foliage), road topology variations, and changing line-of-sight (LOS) conditions. While the classic Hata (1980) model established the foundation for urban path loss prediction, modern V2I systems operate at higher frequencies and in more complex environments, necessitating enhanced modeling approaches. Experimental data indicate that path loss exponents are likely to be approximately 2.0 or higher in LOS scenarios and considerably greater than 3.5 in NLOS situations in dense urban settings (Abbas et al., 2013). This observation aligns with the seminal work by Chang and Yang (1997), who established the fundamental relationship between urban morphology and path loss exponent variations. To better model LOS-to-NLOS transitions, hybrid approaches like two-slope models have been proposed (Fernández et al., 2024).

While deterministic devices such as 3D ray tracing are more effective than others in modeling complex wave interactions such as diffraction and reflection, their computational expense emphasizes the value of balanced techniques with optimized efficiency and accuracy in the design of urban V2I networks (Pätzold and De Nardis, 2020). Fraile et al. (2000) demonstrated that simplified ray tracing models can achieve reasonable accuracy while significantly reducing computational requirements, making them suitable for large-scale network planning applications.

### 2.2 ML path loss prediction

ML has emerged as a powerful tool for improving path loss modeling in wireless communication, offering enhanced accuracy and adaptability over traditional empirical and deterministic models (Goodfellow et al., 2016). ML techniques have been widely applied in various domains, including image recognition (Krizhevsky et al., 2012), natural language processing (Devlin et al., 2019), and wireless communication systems (Zhang et al., 2019). Path loss prediction, a crucial component of wireless network planning, is inherently a regression problem, making it well-suited for supervised learning techniques such as support vector machines, artificial neural networks (ANN), random forests, and K-nearest neighbors (Murphy, 2012). Among these, ANN models have demonstrated superior accuracy in path loss estimation compared to empirical models (Bishop, 2006). For instance, Zhang et al. (2019) developed a real-time channel prediction model that estimates path loss (PL) and packet drop probability in Dedicated Short-Range Communications (DSRC) systems, highlighting the flexibility of ML approaches in dynamic vehicular environments.

The adaptability of ML in path loss modeling is attributed to its ability to learn directly from measured propagation data, allowing models to generalize better across varying environments (Oroza et al., 2017). This approach addresses the limitations of traditional channel models as identified by Molisch et al. (2011), who emphasized the need for context-aware propagation modeling in vehicular communications. Recent comprehensive studies by Huang et al. (2022a) have demonstrated that ML models can effectively capture the complex relationship between environmental features and path loss in V2I scenarios. The follow-up work by Huang et al. (2022b) further validated these findings through extensive field measurements across diverse urban environments. Unlike traditional models that rely on predefined propagation assumptions, ML-based approaches use extensive datasets collected from real-world conditions to train algorithms for accurate signal prediction. Furthermore, ML models incorporate key radio environment factors, including distance, frequency, antenna height, terrain type, and obstacles, to enhance predictive accuracy (Uccellari et al., 2016).

### 2.3 Deep learning for path loss prediction

Deep learning, a subset of ML characterized by neural networks with multiple hidden layers, has shown promising results in path loss prediction. Deep neural networks (DNNs) can automatically extract features from raw data, eliminating the need for manual feature engineering (Tarhuni and Ouni, 2022). This capability is particularly valuable in complex urban environments where signal propagation is influenced by numerous factors. For example, Thrane et al. (2020) demonstrated that DNNs outperform traditional path loss models in urban scenarios by capturing intricate relationships between environmental variables and signal attenuation (Gho et al., 2019).

Recurrent Neural Networks and Long Short-Term Memory networks have been applied to path loss prediction in dynamic environments, leveraging their ability to model temporal dependencies in signal propagation (Pätzold and De Nardis, 2020). These approaches are especially relevant for V2I communications, where both the vehicle and the surrounding environment may change rapidly. Additionally, Convolutional Neural Networks have been utilized to process spatial information from environmental maps, enabling more accurate path loss predictions in urban settings (Filippi and Bazzi, 2021).

Despite their advantages, deep learning approaches face challenges such as the need for large training datasets, computational complexity, and potential overfitting (Gozalvez and Sepulcre, 2022). Wu et al. (2020) proposed a novel transfer learning approach to address these limitations by leveraging knowledge from source domains with abundant data to improve prediction accuracy in target domains with limited data. Hybrid models that combine deep learning with traditional path loss models have been proposed to address these limitations, offering a balance between accuracy and computational efficiency (Huang et al., 2022a).

## 3 Methodology

**Figure 1:**
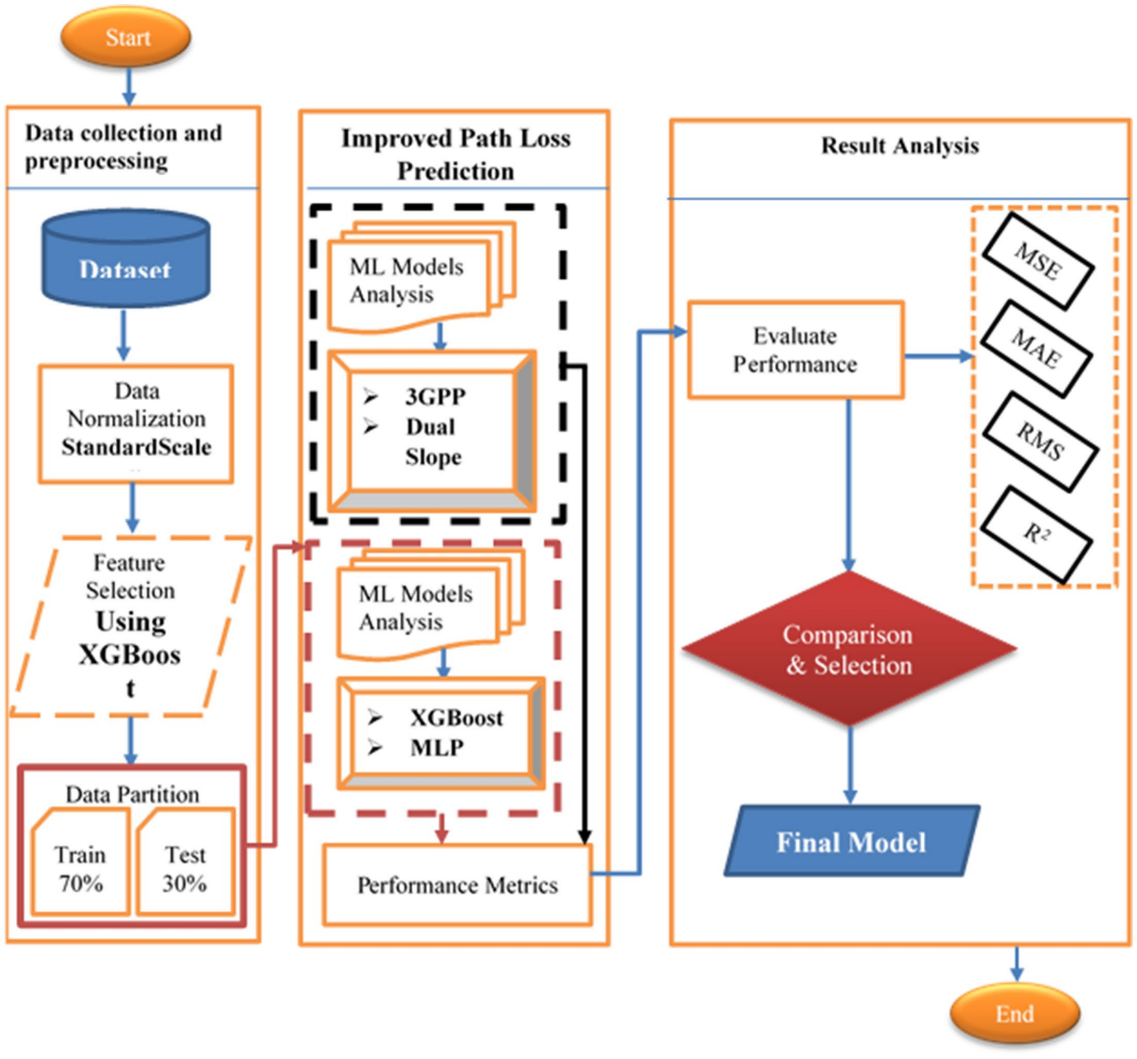
The workflow of this study.

The methodology of this study is designed to systematically evaluate and enhance path loss prediction for V2I communications in dynamic urban environments. Guided by the workflow illustrated in Figure 1, this section outlines a structured three-phase approach: (i) data collection and preprocessing, (ii) model development and analysis, and (iii) performance evaluation. The objective is to validate the effectiveness of ML models compared to traditional empirical approaches, while addressing the challenges posed by critical urban variables such as distance, obstruction density, and antenna height.

The study begins with the acquisition of real-world data across diverse urban scenarios, ensuring robust representation of signal propagation dynamics. This data undergoes rigorous preprocessing including normalization and feature selection to enhance model generalizability. Subsequently, state-of-the-art ML models (XGBoost and MLP) are developed and evaluated against empirical frameworks (3GPP TR 38.901 and Dual Slope) to identify the optimal prediction method for urban V2I networks. By aligning with the workflow in Figure 1, the proposed methodology ensures reproducibility, minimizes bias, and promotes adaptability to real-world complexities ultimately supporting the development of reliable 5G/6G vehicular communication systems.

### 3.1 Dataset collection

We utilize an open-access dataset (30), which was gathered under a V2I measurement campaign in Bologna. Eight RSU positions as transmitters (Tx) are included in this dataset, which function in between 5.9 GHz. The transmitters were mounted at two different heights: 6.5 and 10.5 m. The receiver (Rx) was mounted on the roof of a car at a height of 1.75 m. The dataset includes RSSI measurements, which were converted to path loss using the formula shown in Equation 1:


(1) $$\mathrm{PL}=P_{\mathrm{Tx}}-P_{\mathrm{Rx}}+L_{\text{cable}}-G_{\mathrm{Tx}}-G_{\mathrm{Rx}}\;$$

where $P_{\mathrm{Tx}}$ is the transmission power, and $P_{\mathrm{Rx}}$ is the received power, which in this context is the measured *RSSI* value, $L_{\text{cable}}$ *r*epresents the cable loss, and $G_{\mathrm{Tx}}$ and $G_{\mathrm{Rx}}$ are the *Tx* and *Rx* antenna gains, respectively.

The dataset includes measurements from various urban environments, including open areas, narrow streets, and areas with dense buildings. GPS coordinates for both transmitters and receivers were recorded, allowing for accurate distance calculations and environmental classification.

### 3.2 Data preprocessing

To ensure data quality and reliability prior to model training, several preprocessing techniques were applied to the raw V2I communication dataset. These techniques were designed to handle noise, normalize feature scales, and preserve the statistical distribution of environmental classes across training, validation, and testing sets.

#### 3.2.1 Outlier elimination

Outliers were identified and removed using the Interquartile Range (IQR) method. For a given feature X, the IQR is calculated as shown in Equation 2:


(2) $$\mathrm{IQR}=Q_{3}-Q_{1}$$

where $Q_{1}$ and $Q_{3}$ are the first and third quartiles, respectively. A data point x is considered an outlier if $x\langle Q_{1}-1.5*\mathrm{IQR}\;\text{or}\;x\rangle Q_{3}+1.5*\mathrm{IQR}.$

#### 3.2.2 Feature scaling

Min-max normalization was used to rescale all numerical features to a standard range [0, 1]. For each feature value x, the normalized value x′ is computed as:


$$x^{'}=(x-\min(x))/(\max(x)-\min(x))$$

This ensures that all input features contribute proportionally during model training.

#### 3.2.3 Temporal averaging

To reduce temporal noise in RSSI measurements, a sliding window average was applied. For a signal *s*(*t*) over time, the smoothed signal s−(t) is given by Equation 3:


(3) s−(t)=(1/N)∗Σ_{i=0}^{N−1}s(t−i)

where *N* is the window size.

Finally, the dataset was split into three subsets: 80% for training, 10% for validation, and 10% for testing. Stratified sampling was used to maintain the proportional distribution of environmental classes (open, suburban, and dense urban) across all subsets, ensuring fair evaluation of model performance.

### 3.3 Feature importance analysis

**Figure 2:**
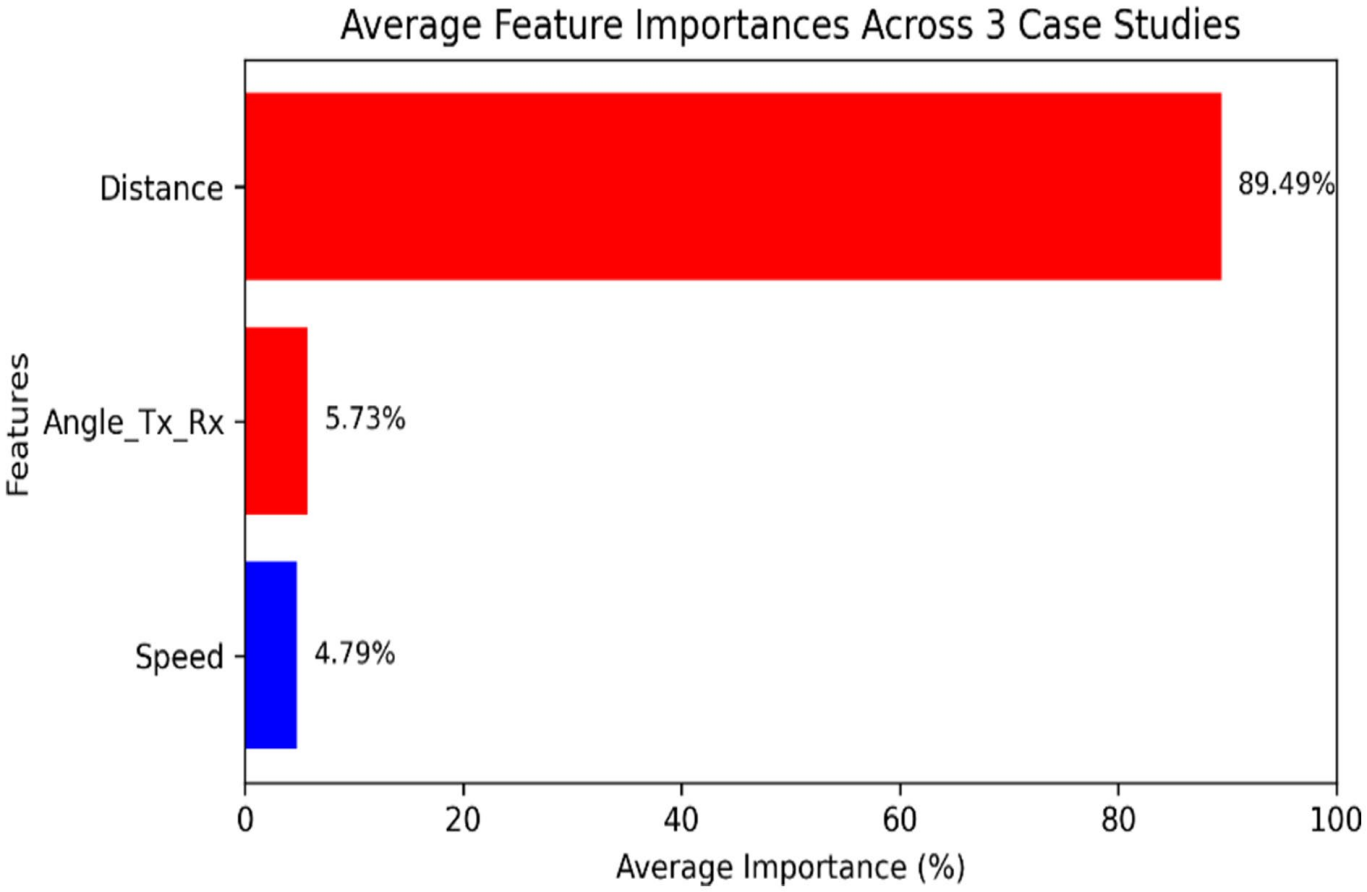
Feature importance in XGBoost model.

Feature importance analysis was conducted to identify the most significant factors affecting path loss prediction accuracy. Figure 2 shows the relative importance of different features in the XGBoost model.

The analysis reveals that distance, environmental class, and transmitter height are the most significant factors affecting path loss prediction accuracy. While receiver latitude and longitude also show high importance, this is primarily because they implicitly capture the road network geometry and building distribution patterns in our study areas.

Regarding the use of latitude and longitude as features, we acknowledge that an intrinsic coordinate system (such as a polar coordinate system with radius = distance and angle with origin at the RSU might be theoretically more elegant. However, we found that latitude and longitude provided practical advantages in our specific dataset, as they inherently encode spatial relationships between the transmitter and receiver within the urban landscape. Future work could explore alternative coordinate systems to potentially improve model performance further. The most critical hyperparameters include in Table 1.

**Table 1:** XGBoost various parameters.

Hyperparameter	Value
Number of boosting rounds	500
Learning rate (shrinkage)	0.05
Maximum depth of trees	6
Minimum sum of instance weight	5
Minimum loss reduction	0.1
Subsample ratio of training data	0.8

### 3.4 Environmental classification

We classify the urban environments into three categories based on building density, street layout, and vegetation coverage:

- *Open Urban Environment:* Characterized by wide streets, low building density (<50 buildings/km^2^), and minimal vegetation. These areas typically have excellent line-of-sight (LOS) conditions between the RSU and vehicles.
- *Suburban Environment:* Medium building density (50–200 buildings/km^2^), moderate street widths, and variable vegetation. These areas feature a mix of LOS and non-line-of-sight (NLOS) conditions.
- *Dense Urban Environment:* High building density (>200 buildings/km^2^), narrow streets, and urban canyons. These areas predominantly have NLOS conditions with significant multipath effects. The classification methodology builds upon the urban propagation environment categorization proposed by Neskovic et al. (2001), who established the correlation between building density metrics and radio propagation characteristics.

This classification system provides a structured approach to understanding how different urban characteristics affect signal propagation and path loss prediction.

### 3.5 Path loss prediction models

**Figure 3:**
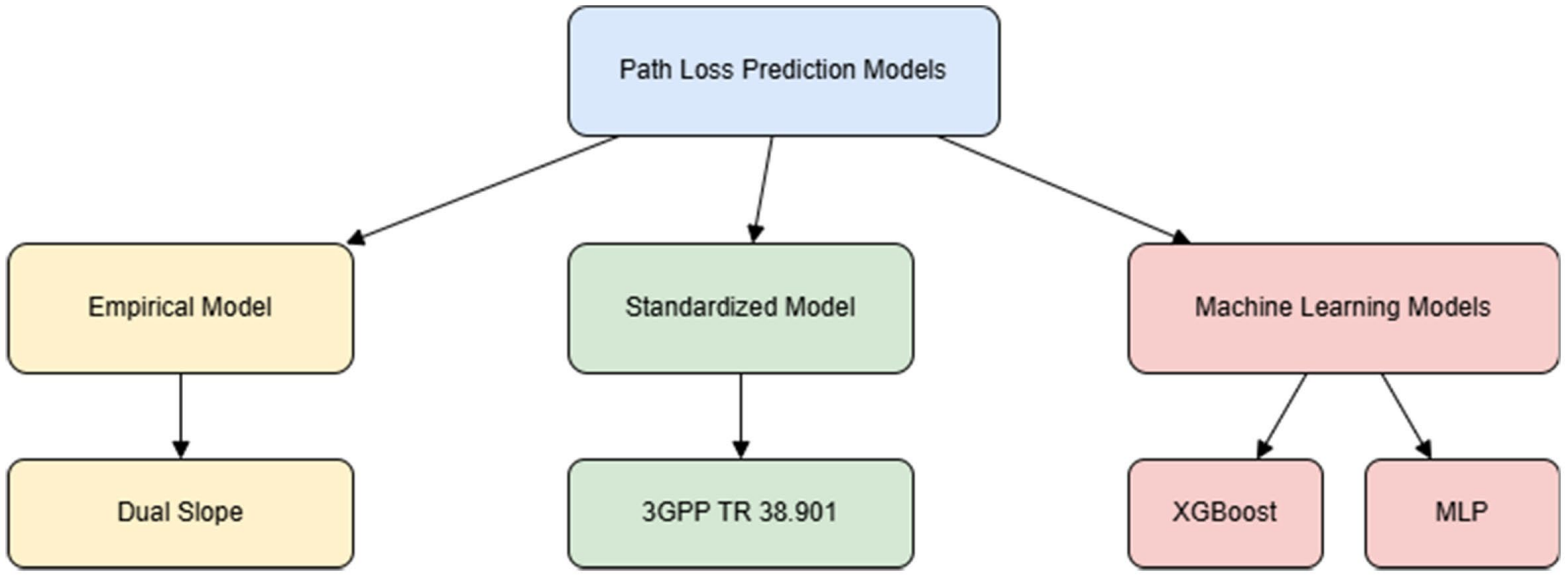
Block diagram of evaluated path loss prediction models categorized by modeling approach.

This section presents a comprehensive overview of the four path loss prediction models evaluated in this study, each representing a different class of modeling approaches. As shown in Figure 3, we begin with the dual slope model, an empirical formulation known for its simplicity and effectiveness in capturing distinct propagation characteristics at varying distance intervals. This is followed by the 3GPP model, a standardized model widely adopted in cellular communication research. The last two models, XGBoost and MLP, belong to the machine learning domain and leverage data-driven training to enhance prediction accuracy. Together, these models provide a diverse analytical foundation for evaluating path loss behavior in V2I communication scenarios.

#### 3.5.1 Dual slope model

The Dual Slope model is an empirical approach that accounts for distinct propagation characteristics at different distances. It is mathematically represented in Equation 4:


(4) $$\begin{array}{l} \mathrm{PL}(d)=\\ \left\{ \begin{array}{cc} PL_{0}+n_{1}\log_{10}(d/d_{0}), & d<d_{\text{break}}\\ PL_{0}+n_{1}\log_{10}(d_{\text{break}}/d_{0})+n_{2}\log_{10}(d/d_{\text{break}}), & d\geq d_{\text{break}} \end{array}\right. \end{array}$$

where: PL(d) represents the predicted path loss at distance d ; $PL_{0}$ denotes the reference path loss at $d_{0}$ ; $d_{\text{break}}$ is the breakpoint distance; $n_{1}$ and $n_{2}$ are the path loss exponents before and after $d_{\text{break}}$ .

The parameters $PL_{0}$ , $d_{\text{break}}$ , $n_{1}$ , and $n_{2}\;$ were individually estimated for each case study using a least-squares curve-fitting approach, minimizing the error between the model’s predictions and the measured path loss data.

Several studies (32–34) have explored the effectiveness of the Dual Slope model in different environments.

#### 3.5.2 3GPP model

The 3GPP model is a standardized path loss model developed by the 3rd Generation Partnership Project for various wireless communication scenarios. For urban V2I communications at 5.9 GHz, the model is defined in Equation 5:


(5) $$\begin{array}{l} \mathrm{PL}(d)=40*\log_{10}\;(d)+7.8-18*\log_{10}(h_{\mathrm{BS}})\\ -18*\log_{10}\;(h_{\mathrm{MS}})+2*\log_{10}\;(\frac{f_{c}}{5.0}) \end{array}$$

where *d* is the distance between the transmitter and receiver in kilometers, $\;h_{\mathrm{BS}}$ is the base station (RSU) height in meters, $h_{\mathrm{MS}}$ is the mobile station (vehicle) height in meters, and $f_{c}$ is the carrier frequency in GHz.

#### 3.5.3 XGBoost model

XGBoost is an ensemble learning method that uses a gradient boosting framework to build a collection of decision trees. The model was implemented with the following hyperparameters:

Number of estimators: 100
Maximum depth: 6
Learning rate: 0.1
Subsample ratio: 0.8
Column sample by tree: 0.8
Minimum child weight: 1
Regularization alpha: 0
Regularization lambda: 1

The total number of learnable parameters in our XGBoost model is approximately 6,500, varying slightly based on the specific tree structures learned during training. Mean squared error was used as the loss function during training.

#### 3.5.4 MLP model

The MLP is a feedforward artificial neural network with multiple layers of nodes. Our implementation consists of:

Input layer: 8 neurons (corresponding to our feature set)
Hidden layer 1: 64 neurons with ReLU activation
Hidden layer 2: 32 neurons with ReLU activation
Hidden layer 3: 16 neurons with ReLU activation
Output layer: 1 neuron with linear activation

Additional architectural details:

- Dropout rate of 0.2 between layers to prevent overfitting
- Batch normalization after each hidden layer
- Adam optimizer with learning rate of 0.001
- Batch size of 32
- Early stopping with patience of 10 epochs
- Mean squared error as the loss function

#### 3.5.5 Performance metrics

To evaluate the accuracy and reliability of both models, several performance metrics were computed:

- *Mean Squared Error (MSE):* Measures the average squared differences between predicted and actual values as shown in Equation 6:

(6) $$\mathrm{MSE}=\frac{1}{n}\sum_{i=1}^{n}{\left(y_{i}-\hat{y_{i}}\right)}^{2}$$

where $y_{i}$ is the actual path loss ${\hat{y}}_{i}$ is the predicted path loss, and *n* is the number of observations.

- *RMSE*: Provides the standard deviation of residuals, indicating model precision as defined in Equation 7:

(7) $$\text{RMSE}=\sqrt{\frac{1}{n}\sum_{i=1}^{n}{\left(y_{i}-{\hat{y}}_{i}\right)}^{2}}$$

- *R-squared (R^2^):* measures the proportion of variance in the target variable explained by the model as shown in Equation 8, providing an overall indication of model fit. An R^2^value close to 1 suggests that the model accounts for most of the variance and makes accurate predictions. A value near 0 implies the model performs no better than simply predicting the mean of the observed data. Negative values indicate the model performs worse than this naive, mean-based prediction.

(8) $$R2\text{Score}=1-\frac{\sum_{i=1}^{n}{\left(y_{i}-\hat{y_{i}}\right)}^{2}}{\sum_{i=1}^{n}{\left(y_{i}-\bar{y}\right)}^{2}}$$

- *Mean Absolute Error (MAE):* Represents the average absolute differences between predicted and actual values as defined in Equation 9:

(9) MAE=1n∑i=1n∣yi−yi^∣

- *Standard Deviation of Residuals (Std Dev):* Measures the spread of errors in prediction, indicating model consistency in Equation 10:

(10) $$\sigma =\sqrt{\frac{1}{n}\sum_{i=1}^{n}{\left(y_{i}-{\hat{y}}_{i}-\bar{e}\right)}^{2}}$$

where $\bar{e}$ is the mean error.

These metrics provide a comprehensive evaluation of the models’ predictive capabilities, with lower error values indicating higher accuracy.

## 4 Results and discussion

This section gives a clear picture of how different path loss prediction models perform in three case studies. Every case study represents a variant V2I communication situation. The XGBoost model is compared with the Dual Slope model, the 3GPP TR 38.901 model, and the MLP. The assessment utilizes benchmark metrics to verify the performance of the models, which include RMSE, MSE, MAE, and R^2^, as presented in Table 2. The findings confirm that machine learning models are more accurate in outcome prediction, particularly XGBoost, with consistently lower error rates and higher R^2^ values across all scenarios. This performance gain is especially evident in dynamic and complex urban environments, where the conventional models are likely to fail to capture the non-linear and non-stationary characteristics of radio wave propagation. These findings point out the potential of data-driven approaches for enhancing the accuracy and robustness of V2I channel modeling in future wireless systems.

**Table 2:** Performance metrics comparison: case studies.

Case study	Model	MSE (dB)	MAE (dB)	RMSE (db)	R^2^
1	3GPP (TR 38.901)	261.71	13.05	16.18	−3.47
	Dual slope	21.86	3.70	4.68	0.61
	MLP	0.19	0.34	0.43	0.83
	XGBoost	0.06	0.19	0.25	0.94
2	3GPP (TR 38.901)	150.97	11.11	12.29	−1.32
	Dual slope	30.83	4.51	5.55	0.52
	XGBoost	0.09	0.20	0.29	0.92
	MLP	0.17	0.30	0.41	0.83
3	3GPP (TR 38.901)	220.25	12.85	14.84	−0.72
	Dual slope	46.13	5.53	6.79	0.64
	XGBoost	0.05	0.16	0.22	0.95
	MLP	0.14	0.28	0.37	0.86

### 4.1 Case study 1: open area

**Figure 4:**
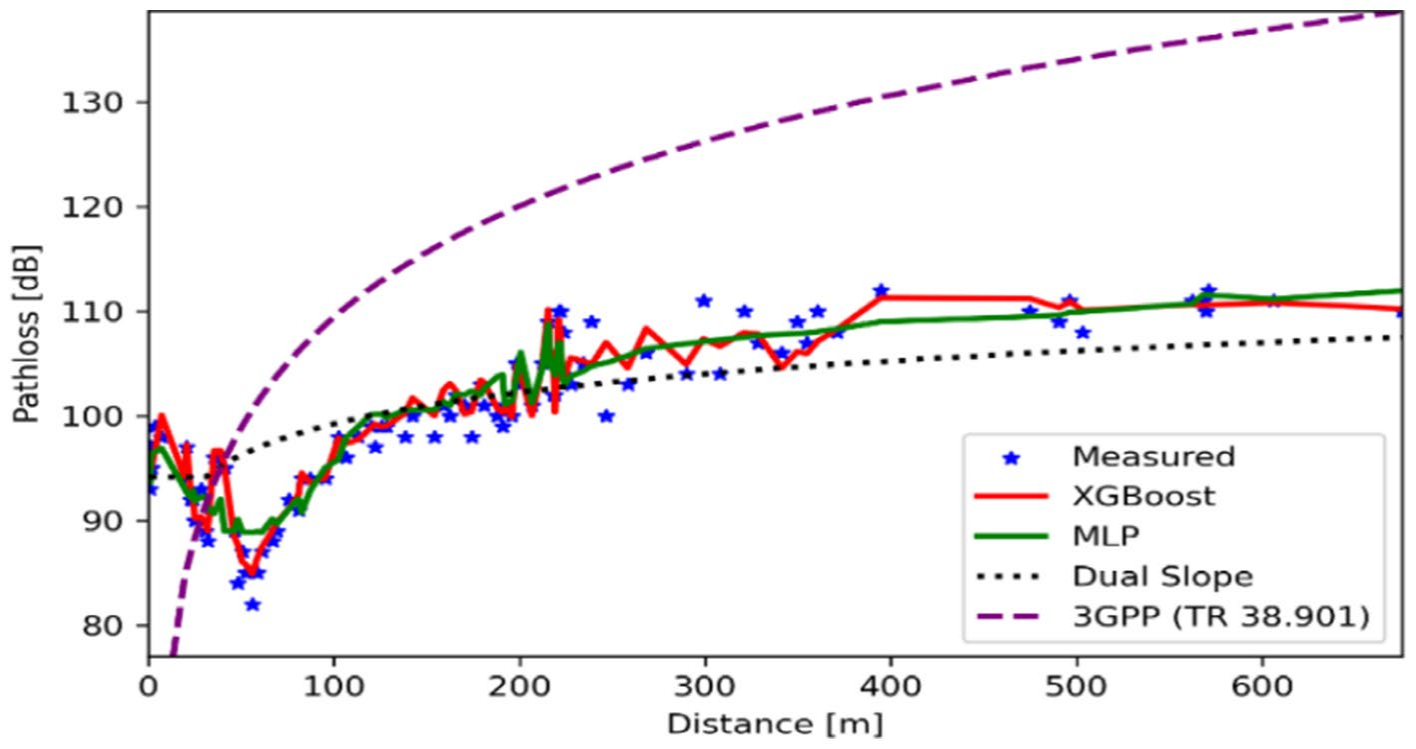
Path loss vs. distance for different prediction models in open urban environment.

Figure 4 illustrates the path loss behavior in an open area with minimal obstructions and compares the performance of machine learning models with traditional approaches. XGBoost performed exceptionally well, achieving an RMSE of just 0.25 dB and an R^2^ of 0.94, outperforming all other models. The MLP model performed well, accurately identifying subtle propagation effects, including ground reflections, with an RMSE of 0.43 dB and an R^2^ of 0.83. In contrast, the traditional models lagged considerably: the Dual Slope model achieved an RMSE of 4.68 dB and an R^2^ of 0.61, whereas the 3GPP TR 38.901 model performed much worse, with an RMSE of 16.18 dB and an R^2^ of −3.47, indicating performance worse than a naive mean-value prediction. These results highlight the shortcomings of static, rule-based models for use in open settings where complicated and multidimensional signal propagation dynamics are the main concern

### 4.2 Case study 2: suburban area

**Figure 5:**
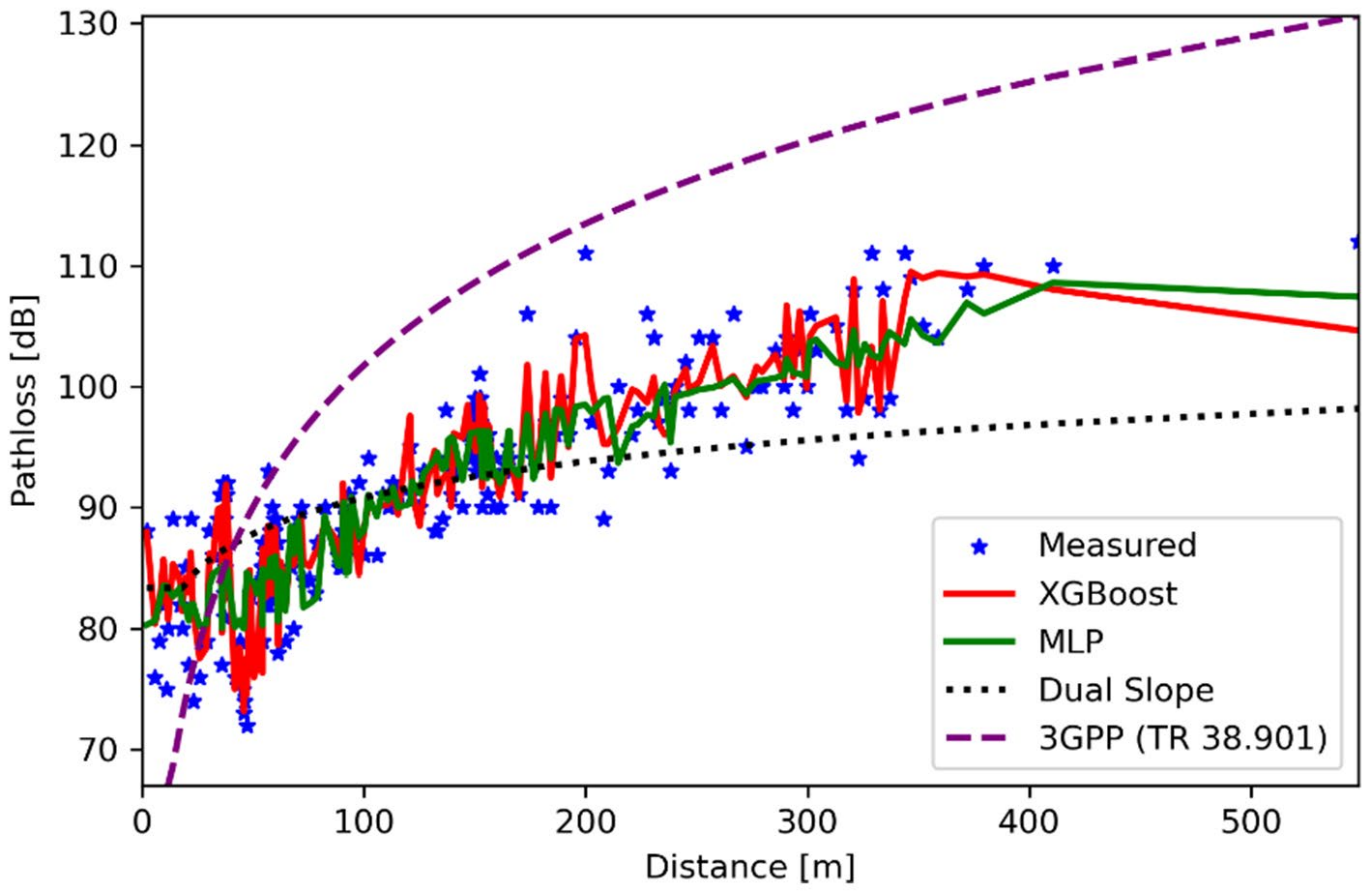
Predicted vs. measured path loss for different models in suburban environment.

Figure 5 illustrates the path loss patterns in a suburban environment with moderate building density and partial obstructions. XGBoost demonstrated the highest accuracy, achieving an RMSE of 0.29 dB and an R^2^ of 0.92, while the MLP model also performed well, with an RMSE of 0.41 dB and an R^2^ of 0.83. In contrast, traditional models showed significantly lower accuracy: the Dual Slope model had an RMSE of 5.55 dB and an R^2^ of 0.52, and the 3GPP TR 38.901 model performed poorly, with an RMSE of 12.29 dB and a negative R^2^ of −1.32. These results highlight the superior adaptability of machine learning models in handling the variability of suburban environments—such as the presence of trees and mid-rise buildings—compared to the static nature of traditional, formula-based models.

### 4.3 Case study 3: dense urban area

**Figure 6:**
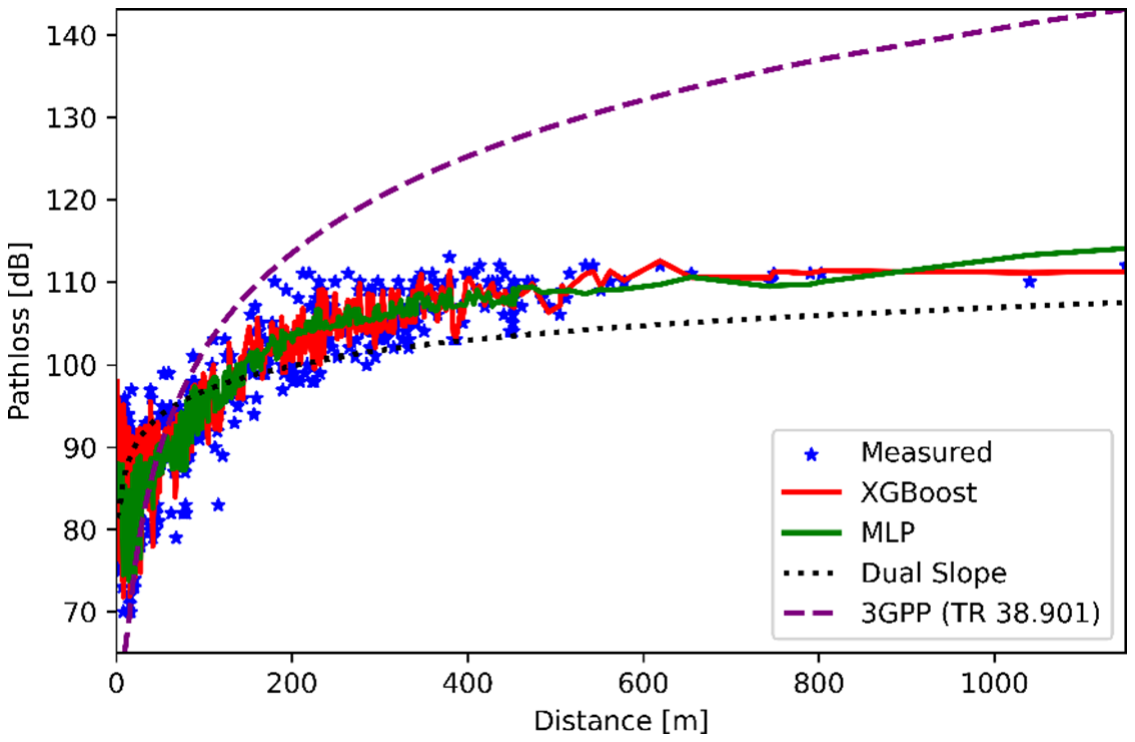
Predicted vs. measured path loss for different models in dense urban.

Figure 6 illustrates path loss behavior in dense urban areas characterized by tall buildings and pronounced multipath effects. XGBoost delivered outstanding performance, achieving an RMSE of 0.22 dB and an R^2^ of 0.95, while the MLP model also performed well, with an RMSE of 0.37 dB and an R^2^ of 0.86. In contrast, traditional models struggled to represent the complexity of the environment: the Dual Slope model recorded an RMSE of 6.79 dB and an R^2^ of 0.64, while the 3GPP TR 38.901 model fared the worst, with an RMSE of 14.84 dB and a negative R^2^ of −0.72. These results underscore XGBoost’s ability to capture nonlinear propagation effects such as shadowing and diffraction and clearly reveal the limitations of static, formula-based models like 3GPP in accurately modeling signal behavior in complex urban settings.

### 4.4 Environmental impact on path loss

Our analysis indicates that environmental factors significantly influence path loss characteristics. Table 3 summarizes the critical variables affecting signal reliability in different urban environments and proposes specific actions for improving connectivity. Gozalvez et al. (2012) similarly observed that environmental context awareness is crucial for optimizing V2I communication systems, particularly in heterogeneous urban settings. In open urban environments, transmission power optimization can significantly improve connectivity. However, in dense urban environments with sharp road curves, increasing transmission power provides minimal benefits, and strategic RSU placement becomes more critical.

**Table 3:** Critical variables and recommended actions for different urban environments.

Environment	Critical variables	Recommended actions
Open urban	Transmission power, antenna height	Optimize transmission power, adjust antenna height based on coverage requirements
Suburban	Vegetation density, road curvature	Increase antenna height above vegetation, strategic RSU placement at road curves
Dense urban	Building density, street width	Deploy multiple RSUs with overlapping coverage, position RSUs at street intersections

## 5 Conclusion

This study demonstrates that machine learning approaches, particularly XGBoost, consistently outperform traditional path loss prediction models in urban V2I communication systems. The superior performance of ML models is attributed to their ability to learn non-linear signal fluctuations and environmental factors directly from empirical data.

Future work could explore hybrid models combining physics-based and data-driven approaches, investigate alternative coordinate systems for feature representation, and extend the analysis to additional frequency bands relevant to emerging V2I communication standards.

## Data Availability

The original contributions presented in the study are included in the article/supplementary material, further inquiries can be directed to the corresponding author.
